# The impact of low-magnitude high-frequency vibration on fracture healing is profoundly influenced by the oestrogen status in mice

**DOI:** 10.1242/dmm.018622

**Published:** 2014-11-07

**Authors:** Esther Wehrle, Astrid Liedert, Aline Heilmann, Tim Wehner, Ronny Bindl, Lena Fischer, Melanie Haffner-Luntzer, Franz Jakob, Thorsten Schinke, Michael Amling, Anita Ignatius

**Affiliations:** 1 Institute of Orthopedic Research and Biomechanics, Center of Musculoskeletal Research, University of Ulm, 89081 Ulm, Germany.; 2 Orthopedic Center for Musculoskeletal Research, Orthopedic Department, University of Würzburg, 97074 Würzburg, Germany.; 3 Institute of Osteology and Biomechanics, University Medical Center Hamburg-Eppendorf, 20246 Hamburg, Germany.

**Keywords:** Whole-body vibration, LMHFV, Fracture healing, Oestrogen receptor signalling, Wnt signalling

## Abstract

Fracture healing is impaired in aged and osteoporotic individuals. Because adequate mechanical stimuli are able to increase bone formation, one therapeutical approach to treat poorly healing fractures could be the application of whole-body vibration, including low-magnitude high-frequency vibration (LMHFV). We investigated the effects of LMHFV on fracture healing in aged osteoporotic mice. Female C57BL/6NCrl mice (*n*=96) were either ovariectomised (OVX) or sham operated (non-OVX) at age 41 weeks. When aged to 49 weeks, all mice received a femur osteotomy that was stabilised using an external fixator. The mice received whole-body vibrations (20 minutes/day) with 0.3 ***g*** peak-to-peak acceleration and a frequency of 45 Hz. After 10 and 21 days, the osteotomised femurs and intact bones (contra-lateral femurs, lumbar spine) were evaluated using bending-testing, micro-computed tomography (μCT), histology and gene expression analyses. LMHFV disturbed fracture healing in aged non-OVX mice, with significantly reduced flexural rigidity (−81%) and bone formation (−80%) in the callus. Gene expression analyses demonstrated increased oestrogen receptor β (ERβ, encoded by *Esr2*) and *Sost* expression in the callus of the vibrated animals, but decreased β-catenin, suggesting that ERβ might mediate these negative effects through inhibition of osteoanabolic Wnt/β-catenin signalling. In contrast, in OVX mice, LMHFV significantly improved callus properties, with increased flexural rigidity (+1398%) and bone formation (+637%), which could be abolished by subcutaneous oestrogen application (0.025 mg oestrogen administered in a 90-day-release pellet). On a molecular level, we found an upregulation of ERα in the callus of the vibrated OVX mice, whereas ERβ was unaffected, indicating that ERα might mediate the osteoanabolic response. Our results indicate a major role for oestrogen in the mechanostimulation of fracture healing and imply that LMHFV might only be safe and effective in confined target populations.

## INTRODUCTION

Because adequate mechanical stimuli increase bone mass and improve fracture repair (Claes and Heigele, 1999; Ozcivici et al., 2010), mechanical intervention therapies, including whole-body vibration (WBV), are increasingly used to treat osteoporotic bone loss (Gómez-Cabello et al., 2012). ‘Low-magnitude high-frequency vibration’ (LMHFV) became of interest because many preclinical and clinical studies demonstrated its anabolic effects on healthy and osteoporotic bone (Rubin et al., 2004; Xie et al., 2006; Xie et al., 2008). LMHFV combines very low accelerations of ≤1 ***g*** (***g***, gravitational acceleration, 1 ***g***=9.81 m/s^2^) with a high frequency, between 20 and 90 Hz, inducing extremely small strains of ~5–10 με (strain magnitude, symbol ε) in bone tissue; considerably less than the peak strains generated during activity (which are 2000–3000 με) (Xie et al., 2006). Thus it has been proposed that the mechanical signal driving the osteogenic response of bone cells might be oscillatory acceleration rather than the distortion of the bone matrix (Ozcivici et al., 2010). The mechanisms by which cells recognise and transduce mechanical signals are complex. A network of molecules are involved in mechanotransduction, including oestrogen receptor (ER)-mediated pathways and Wnt/β-catenin signalling (Liedert et al., 2006; Rubin et al., 2006).

Because of its osteoanabolic effects, LMHFV could also be used to treat poorly healing bone fractures. Delayed or incomplete fracture repair is a major issue in orthopaedic surgery, with an incidence of ~10% and a high socioeconomic burden (Tzioupis and Giannoudis, 2007). In particular, osteoporotic fractures are frequently associated with complications, which are partially caused by the decreased regenerative capacity of osteoporotic bone (Giannoudis et al., 2007). However, whereas most authors agree that LMHFV has an osteogenic effect during bone remodelling, its benefit for fracture healing is unclear. Currently, only a few preclinical studies exist, and these report conflicting results (Komrakova et al., 2013; Leung et al., 2009; Stuermer et al., 2010a). Recently, we demonstrated that LMHFV provoked anabolic effects in non-fractured bone, but failed to increase bone formation in fracture repair in the same individual (Wehrle et al., 2014). In that study, we used young non-ovariectomised (non-OVX) mice with high regenerative capacity and oestrogen levels, which poorly reflects osteoporotic fracture healing. Although evidence indicates that LMHFV might stimulate fracture repair, particularly in OVX animals, the underlying mechanism is unclear (Shi et al., 2010). OVX leads to rapidly decreased oestrogen levels and induces bone loss both in humans (Aitken et al., 1973) and rodents (Bouxsein et al., 2005), indicating its crucial role in bone metabolism. Oestrogen acts through ERα and ERβ, which are expressed in osteoblasts, osteocytes and osteoclasts, with different ratios for cortical and trabecular bone, thereby regulating cell activity in a complex fashion (Hall et al., 2001). ERα is considered the most essential receptor for mediating oestrogen effects on bone (Börjesson et al., 2013). ERβ appears to either antagonise ERα actions (‘ying-yang’ theory) or modulate ERα-driven gene transcription; hence, the ERα:ERβ ratio might determine the sensitivity of the cell and its biological responses to oestrogen (Böttner et al., 2014; Lindberg et al., 2003). ERs can also be stimulated by mechanical loading (Galea et al., 2013), with bone adaptation to mechanical loading requiring ERα (Lee et al., 2003). The number and activity of ERα is regulated by oestrogen (Jessop et al., 2001). In contrast, ERβ has been proposed to inhibit bone formation in response to mechanical loading (Saxon et al., 2007; Saxon and Turner, 2006). Because bone mechanoresponsiveness is known to alter under oestrogen deficiency (Jagger et al., 1996; Järvinen et al., 2003), LMHFV could provoke differing effects on fracture healing in OVX animals.

**TRANSLATIONAL IMPACT**

**Clinical issue**

Osteoporosis and the associated impairment in fracture healing are a major socioeconomic burden. Because adequate mechanical stimuli increase bone mass and improve fracture repair, mechanical intervention therapies, including whole-body vibration (WBV), are increasingly used to treat osteoporotic bone loss. Low-magnitude high-frequency vibration (LMHFV) became of interest because many pre-clinical and clinical studies have demonstrated its anabolic effects on healthy and osteoporotic bone. LMHFV combines very low accelerations of ≤1 ***g*** (***g***, gravitational acceleration, 1 ***g***=9.81 m/s^2^) with high frequency (between 20 and 90 Hz) vibrations, which induce extremely small strains (fractional changes in bone length) of approximately 5–10 με (strain magnitude, symbol ε) in bone tissue. However, whereas several works have shown that LMHFV has an osteogenic effect during bone remodelling, the benefit of this treatment for fracture healing is still unclear. Currently, only a few pre-clinical studies exist, and they have reported conflicting results. In order to design vibration protocols that are able to improve age- and osteoporosis-associated impaired fracture healing, it is necessary to better understand the molecular mechanisms that underlie LMHFV-mediated effects.

**Results**

The authors of this study used femur osteotomy in aged mice that were previously subjected to ovariectomy (OVX, which reduced oestrogen levels) to model osteoporosis. LMHFV significantly improved fracture healing in OVX mice but considerably impaired it in aged matched non-OVX mice, indicating a major role of the oestrogen status in the mechanostimulation of fracture healing. Gene expression analyses of homogenates from the fracture callus indicated that the anabolic effects of LMHFV in OVX mice might be mediated by oestrogen receptor α (ERα)-driven gene transcription, whereas the inhibition of bone formation in non-OVX mice appeared to involve signalling through ERβ and inhibition of Wnt/β-catenin signalling (a pathway involved in bone homeostasis). Oestrogen supplementation abolished the anabolic effects of LMHFV in OVX mice, further confirming the crucial role of oestrogen in the mechanostimulation of fracture repair.

**Implications and future directions**

Translating the present results into a clinical setting, LMHFV could be an attractive therapy to stimulate bone healing in osteoporosis-affected individuals. However, the distinct oestrogen-dependent effects reported in this study imply that LMHFV might only be safe and effective in elderly postmenopausal osteoporotic women. To elucidate this, further preclinical studies beyond rodent models are mandatory, and caution must be taken in clinical trials to restrict application of this technique to suitable patients.

Building upon our previous study on young animals (Wehrle et al., 2014), we investigated the effect of LMHFV on fracture healing in aged OVX mice compared with non-OVX mice. This study demonstrated that LMHFV significantly improves fracture healing in OVX mice but considerably impairs it in non-OVX mice. Oestrogen supplementation abolished the anabolic effects of LMHFV in OVX mice, further confirming its crucial role in the mechanostimulation of fracture repair. The anabolic effects in OVX mice might be mediated by ERα-driven gene transcription, whereas the inhibition of bone formation in non-OVX mice appears to involve signalling through ERβ and inhibition of Wnt/β-catenin signalling.

## RESULTS

### OVX-induced impaired fracture healing involves ERβ and inhibition of Wnt/β-catenin signaling

**Fig. 1.**
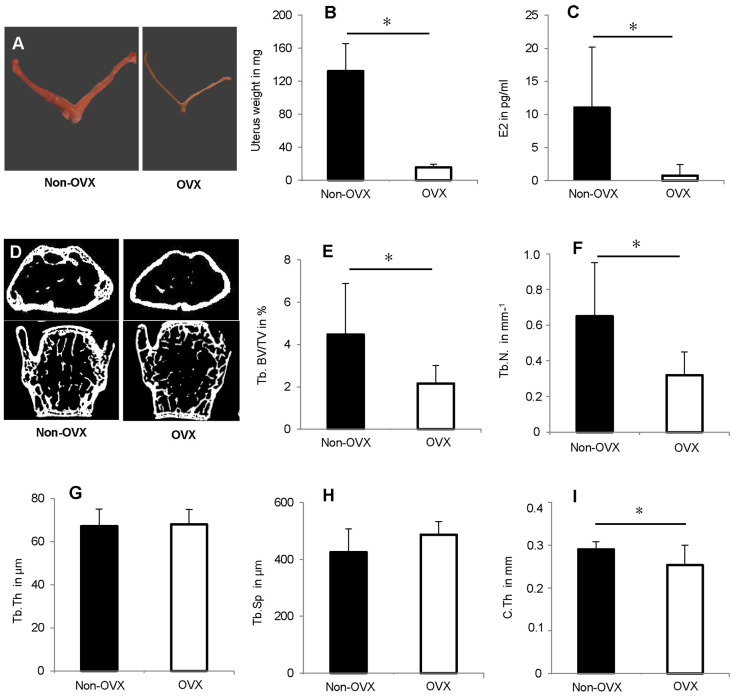
**Effects of OVX on 52-week-old C57BL/6NCrl mice.** OVX was performed at an age of 41 weeks. OVX decreased uterus weight and oestrogen serum levels, and induced an osteopenic phenotype. (A) Images of representative uteri from OVX and non-OVX mice. (B) Uterus weight. *n*=12. (C) Oestrogen serum levels determined using E2-ELISA. *n*=6. (D) Representative μCT sections of the distal femur and the lumbar spine (L6). (E–H) μCT evaluation of trabecular bone (Tb.) in the distal femur, *n*=8. (E) Trabecular bone volume/total volume, Tb. BV/TV; (F) trabecular number, Tb.N; (G) trabecular thickness, Tb.Th; (H) trabecular spacing, Tb.Sp; and (I) cortical thickness in femur diaphysis, C.Th. *P<0.05.

We first analysed the effect of OVX in aged mice to characterise the model (Fig. 1A–I). As expected, OVX induced severe uterus atrophy (Fig. 1A,B) and significantly reduced oestrogen serum levels (Fig. 1C). OVX also induced an osteopenic phenotype. In the trabecular bone of the distal femur, the bone volume density (bone volume over total volume, BV/TV) and trabecular number (Tb.N) were significantly reduced by ~50% (Fig. 1D–F). Trabecular parameters in the lumbar vertebrae (L6) were slightly, but not significantly, influenced [BV/TV: 19±6% OVX, 25±7% non-OVX; Tb.N: 3±1 mm^−1^ OVX, 4±1 mm^−1^ non-OVX; trabecular thickness (Tb.Th): 63±10 μm OVX, 61±6 μm non-OVX; trabecular spacing (Tb.Sp): 256±55 μm OVX, 233±62 μm non-OVX; mean±s.d., Fig. 1D]. In OVX mice, the cortical thickness (C.Th) in the femoral diaphysis was diminished by 14% compared with non-OVX controls (Fig. 1I).

**Fig. 2.**
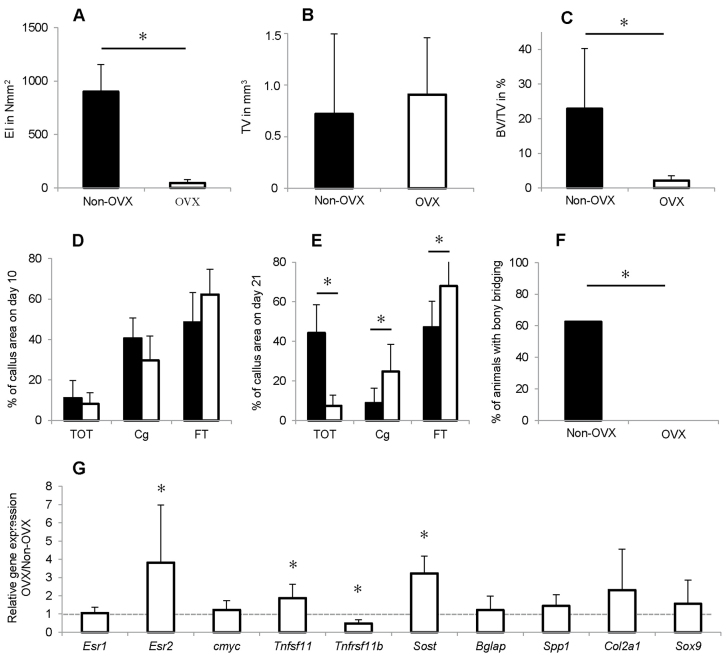
**Effects of OVX on bone healing 10 and 21 days after osteotomy.** OVX significantly impaired fracture healing, increased ERβ expression and inhibited Wnt/β-catenin signalling. (A) Flexural rigidity (EI) of the fracture callus after 21 days. (B) Total callus volume (TV) as assessed by using μCT. (C) Bone volume/total volume (BV/TV) as assessed by using μCT. (D) Callus composition on day 10 as assessed by histomorphometry, given as a percentage of the total callus area. Bone, TOT; cartilage, Cg; and fibrous tissue, FT. (E) Callus composition on day 21 assessed by histomorphometry. (F) Percentage of mice with bony bridging of the fracture gap. (G) Gene expression in OVX versus non-OVX mice as assessed by qPCR. The dashed line indicates the level at which there is no difference in expression. *P<0.05. A–C,E,F, *n*=8; D,G: *n*=5–7.

In fracture healing, OVX significantly reduced the flexural rigidity of the fracture callus (Fig. 2A) and did not alter the total callus volume (Fig. 2B), but significantly diminished the BV/TV by 91% (Fig. 2C). The micro-computed tomography (μCT) evaluation was confirmed by histomorphometry (Fig. 2D,E). Significantly more cartilage (+180%) and fibrous tissue (+44%) were present in OVX mice callus, indicating impaired endochondral ossification with a prolonged presence of cartilage residuals (Fig. 2E). Consistent with these findings, none of the fracture gaps in these mice were completely bridged with bone, all being categorised as ‘not healed’ with ≤2 bridged cortices (Fig. 2F). In contrast, 62% of the non-OVX mice were considered as ‘healed’ with ≥3 bridged cortices at the four assessed locations.

**Fig. 3.**
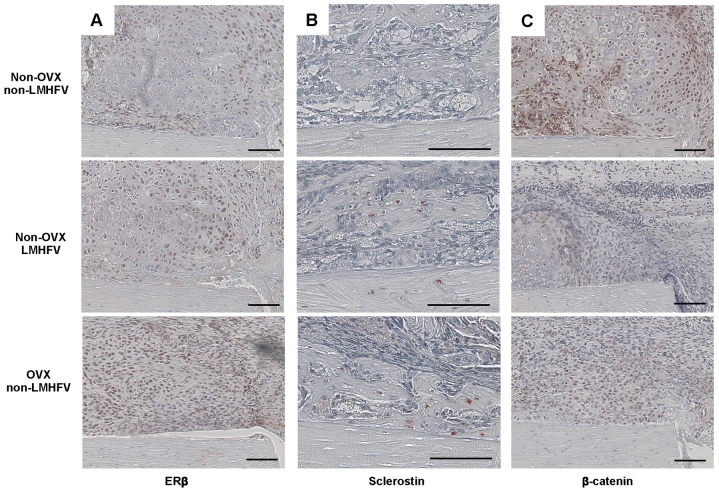
**Representative immunohistological images of the periosteal fracture callus.** Upper row, non-vibrated non-OVX mouse; middle row, vibrated non-OVX mouse; bottom row, non-vibrated OVX mouse. Immunostaining for ERβ (A), sclerostin (B) and β-catenin (C). Scale bars: 100 μm.

Callus homogenate gene expression analysis demonstrated significantly upregulated ERβ (*Esr2*) in the OVX group, whereas ERα (*Esr1*) was unchanged (Fig. 2G). These changes were also apparent at the protein level, as assessed by immunohistochemistry; more fibroblastic and chondroblastic cells were positively stained for ERβ in the fracture callus of the OVX group (Fig. 3A). *Tnfrsf11B*, encoding osteoprotegerin (OPG), was significantly decreased, whereas *Tnsf11*, encoding receptor activator of nuclear factor κB (NFκB) ligand (RANKL), was significantly upregulated after OVX (Fig. 2G), indicating increased osteoclast activity. Furthermore, *Sost*, encoding sclerostin, an inhibitor of the osteoanabolic Wnt signalling pathway, was significantly upregulated. An increased number of sclerostin-positive osteocytes were observed in the newly formed bone in the OVX mice fracture callus (Fig. 3B). Simultaneously, β-catenin, a key molecule of canonical Wnt signalling, was diminished in chondrocytes and osteoblasts in the OVX mice calluses (Fig. 3C), indicating inhibition of Wnt/β-catenin signalling. Other established proliferation and differentiation markers (*cmyc*, *Bglap*, *Spp1*, *Col2a1* and *Sox9*) were not significantly affected by OVX (Fig. 2G).

### LMHFV impaired fracture healing in non-OVX mice but improved it in OVX mice

**Fig. 4.**
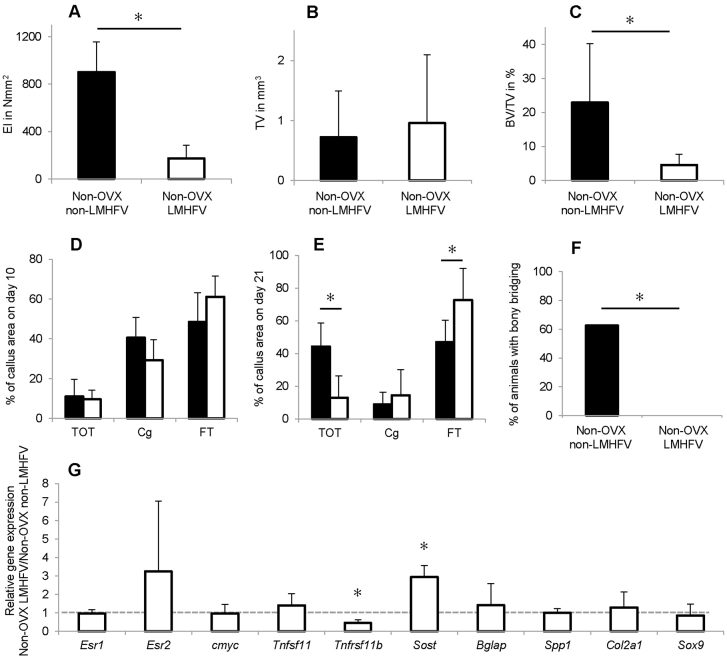
**Effects of LMHFV on bone healing in non-OVX mice at 10 and 21 days after osteotomy.** LMHFV significantly impaired fracture healing, increased ERβ expression and inhibited Wnt/β-catenin signalling. (A) Flexural rigidity (EI) of the fracture callus after 21 days. (B) Total callus volume (TV) as assessed by μCT. (C) Bone volume/total volume (BV/TV) as assessed by μCT. (D) Callus composition on day 10 as assessed by histomorphometry, given as a percentage of the total callus area. Bone, TOT; cartilage, Cg; and fibrous tissue, FT. (E) Callus composition on day 21 as assessed by histomorphometry. (F) Percentage of mice with bony bridging of the fracture gap. (G) Gene expression in vibrated non-OVX mice versus non-vibrated non-OVX mice as assessed by qPCR. The dashed line indicates the level at which there is no difference in expression. *P<0.05. A–C,E,F, *n*=8; D,G: *n*=5–7.

Vibration significantly reduced the flexural rigidity of non-OVX mice callus by 81% compared with the non-vibrated controls (Fig. 4A), as shown by μCT analysis. LMHFV significantly diminished BV/TV by 80% compared with non-vibrated controls, whereas callus volume was unaffected (Fig. 4C,B). Histomorphometry showed that vibration did not significantly alter callus composition on day 10 (Fig. 4D) but led to significantly less bone (−70%) and more fibrous tissue (+55%) on day 21 (Fig. 4E). Consistent with these findings, in the non-OVX mice, none of the fracture gaps were bridged with bone, whereas 62.5% of animals in the non-vibrated group were ‘healed’ (Fig. 4F). Analysis of callus homogenates and immunohistochemistry demonstrated ERβ upregulation in the callus, whereas ERα was unaffected (Fig. 4G; Fig. 3A). Furthermore, *Tnfrsf11B* was significantly downregulated in the vibrated non-OVX mice. *Sost* gene expression was significantly increased, and an increase at the protein level was confirmed by immunohistochemistry. There were more sclerostin-positive osteocytes in the callus and also in the adjacent cortex of the vibrated non-OVX mice (Fig. 3B), indicating diminished Wnt signalling, further confirmed by decreased β-catenin staining of chondrocytes and osteoblasts (Fig. 3C).

**Fig. 5.**
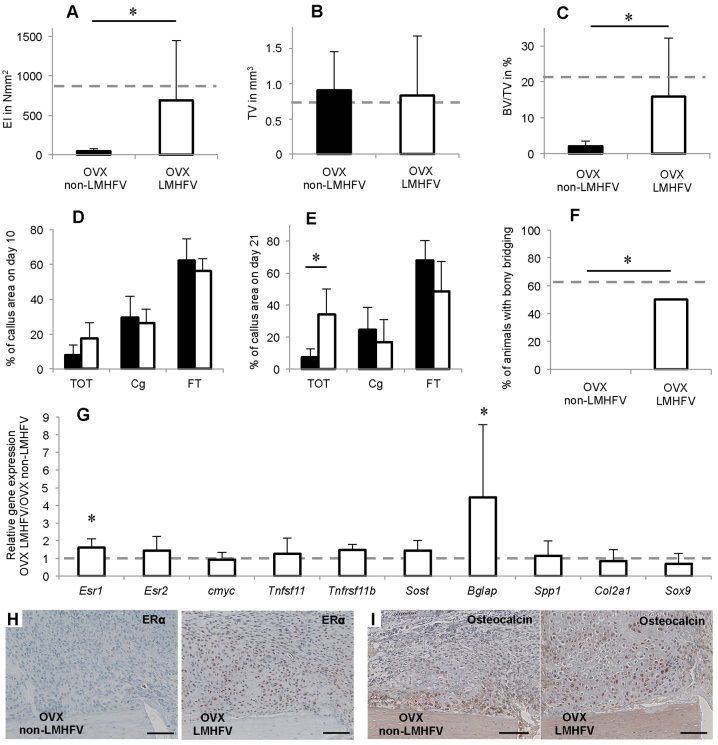
**Effects of LMHFV on bone healing in OVX mice 10 and 21 days after osteotomy.** LMHFV significantly improved fracture healing and increased ERα (*Esr1*) and *Bglap* expression. The dashed lines indicate the values of aged-matched non-OVX mice. (A) Flexural rigidity (EI) of the fracture callus after 21 days. (B) Total callus volume (TV) as assessed by μCT. (C) Bone volume/total volume (BV/TV) as assessed by μCT. (D) Callus composition on day 10 as assessed by histomorphometry, given as a percentage of the total callus area. Bone, TOT; cartilage, Cg; and fibrous tissue, FT. (E) Callus composition on day 21 as assessed by histomorphometry. (F) Percentage of mice with bony bridging of the fracture gap. (G) Gene expression in vibrated OVX versus non-vibrated OVX mice as assessed by qPCR, *n*=6. The dashed line indicates the level at which there is no difference in expression. (H,I) Representative immunohistological images of the periosteal fracture callus of non-vibrated and vibrated OVX mice. Immunostaining for ERα (H) and osteocalcin (I). Scale bars: 100 μm. *P<0.05. A–C,E,F, *n*=8; D,G, *n*=5–7.

In contrast, LMHFV in the OVX mice significantly increased the mechanical performance of the callus (Fig. 5A) and the BV/TV (+637%) compared with non-vibrated mice (Fig. 5C), whereas the total callus volume was unchanged (Fig. 5B). Histomorphometry (Fig. 5D,E) confirmed these results, showing an increased callus bone content of the vibrated OVX mice on day 21 (Fig. 5E). Evaluation of the osteotomy gap osseous bridging categorised 50% of the vibrated OVX mice as healed, whereas no complete cortical bridging in the non-vibrated group was found (Fig. 5F). On the molecular level, LMHFV induced slight, but significant, upregulation of ERα gene (*Ers1*) expression in the OVX mice callus (Fig. 5G), with an increase at the protein level confirmed by immunohistochemistry. ERα staining was increased in fibroblasts, chondrocytes and osteoblasts (Fig. 5H). The most prominent upregulation was that of *Bglap*, encoding osteocalcin (Fig. 5G), indicating increased osteogenic differentiation. Immunohistochemistry of the callus of the non-vibrated OVX mice showed osteocalcin staining mainly in the newly mineralised tissue along the cortex, whereas in the vibrated OVX mice, osteocalcin was also present in the cartilaginous areas (Fig. 5I).

### Oestrogen supplementation improved fracture healing but reduced the anabolic effects of LMHFV in OVX mice

**Fig. 6.**
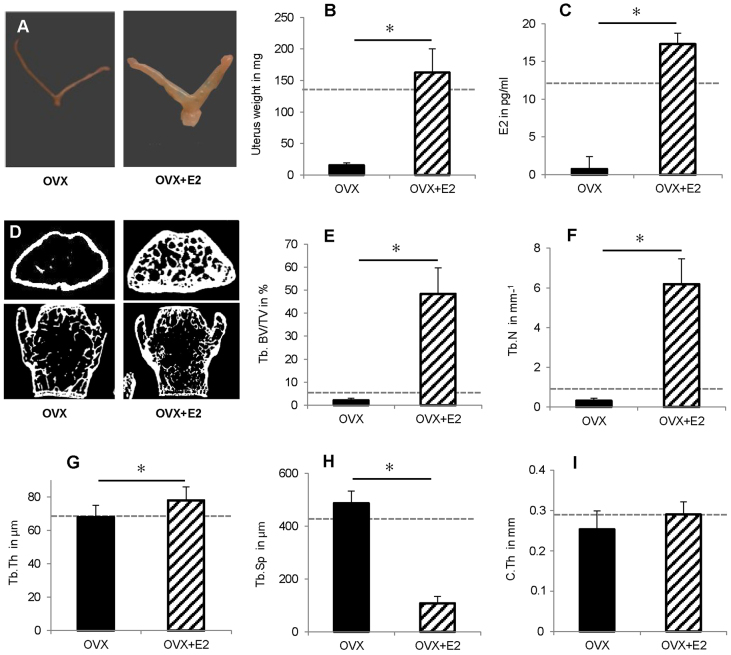
**Effects of oestrogen (E2) supplementation on 52-week-old OVX mice.** OVX and E2 pellet implantation were performed at an age of 41 weeks. E2 increased uterus weight and oestrogen serum levels, and reversed the osteopenic phenotype of non-supplemented OVX mice. The dotted lines indicate the values of aged-matched non-OVX mice. (A) Images of representative uteri. (B) Uterus weight. *n*=7–12. (C) Oestrogen serum levels determined using E2-ELISA. *n*=6. (D) Representative μCT sections of the distal femur and the lumbar spine (L6). (E–H) μCT evaluation of trabecular bone (Tb.) in the distal femur. *n*=8. (E) Trabecular bone volume/total volume, Tb. BV/TV; (F) trabecular number, Tb.N; (G) trabecular thickness, Tb.Th; (H) trabecular spacing, Tb.Sp; and (I) cortical thickness, C.Th. *P<0.05.

Oestrogen supplementation (denoted OVX+E2) reversed the severe uterus atrophy (Fig. 6A,B) and restored the oestrogen serum levels (Fig. 6C) of OVX mice to the physiological levels of non-OVX mice, indicated by dotted lines in Fig. 6. As expected, oestrogen supplementation also induced an anabolic effect on femoral trabecular and cortical bone of the OVX mice, with significantly increased BV/TV, Tb.N and Tb.Th, significantly decreased Tb.Sp and increased C.Th (Fig. 6D–I). The physiological values of age-matched non-OVX mice were far exceeded in the trabecular compartment of the femur (Fig. 1D–H; dotted lines in Fig. 6D–H). In contrast, oestrogen treatment only slightly affected the trabecular bone in the lumbar spine (BV/TV: 26±4% OVX+E2, 19±6% OVX; Tb.N: 4±1 mm^−1^ OVX+E2, 3±1 mm^−1^ OVX; TbTh: 71±7 μm OVX+E2, 63±10 μm OVX; TbSp: 237±49 μm OVX+E2, 256±55 μm OVX; mean±s.d.; Fig. 6D).

**Fig. 7.**
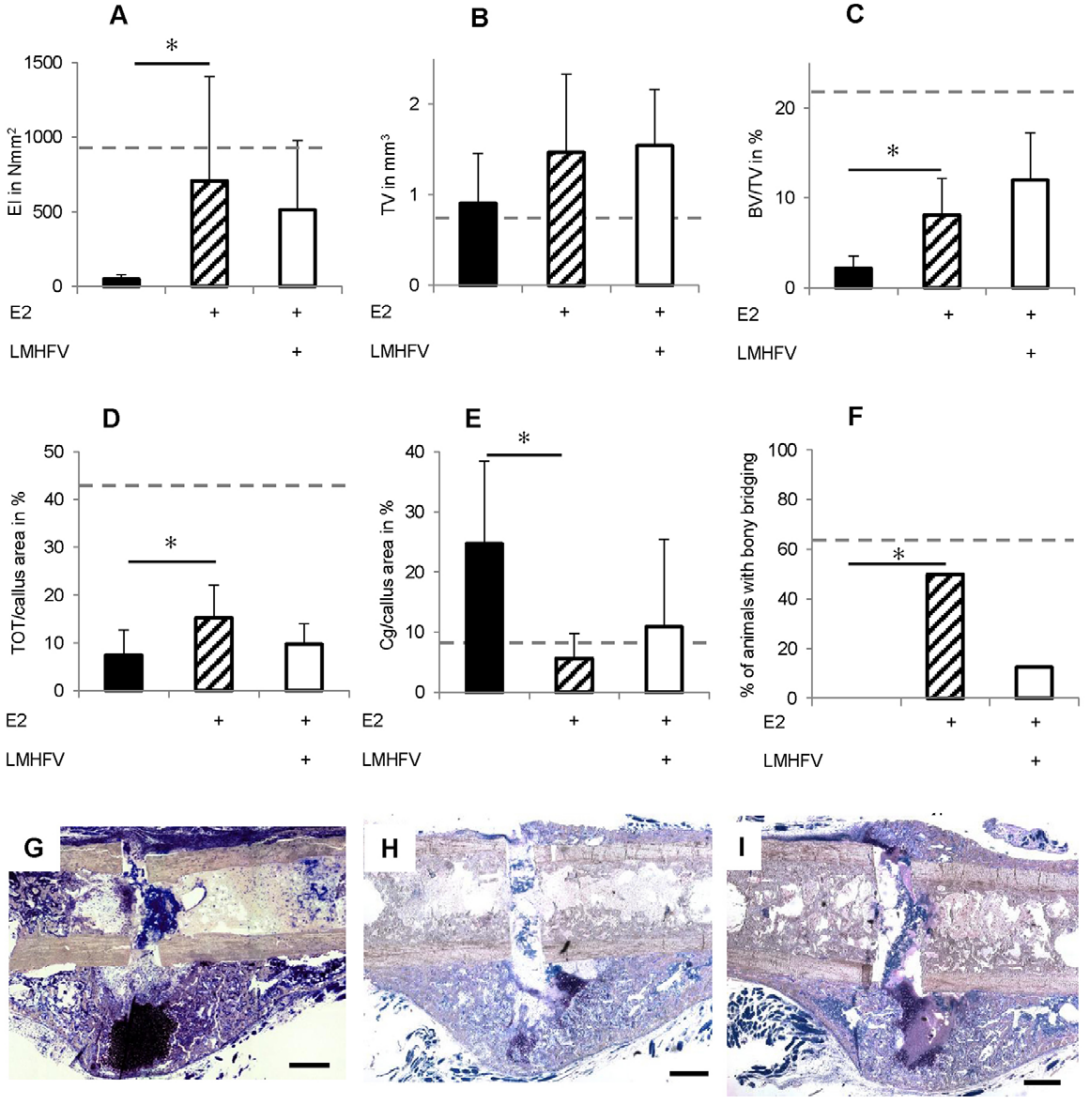
**Effects of LMHFV and oestrogen (E2) supplementation on bone healing in OVX mice 21 days after osteotomy.** The dotted lines indicate the values of aged-matched non-OVX mice. E2 supplementation of OVX mice significantly improved fracture healing. Additional LMHFV did not further increase bone healing. Physiological values of aged-matched non-OVX mice were not achieved. (A) Flexural rigidity (EI) of the fracture callus after 21 days. (B) Total callus volume (TV) as assessed by μCT. (C) Bone volume/total volume (BV/TV) assessed using μCT. (D) Callus composition on day 10 assessed by histomorphometry, given as a percentage of the total callus area. Bone, TOT; cartilage, Cg; and fibrous tissue, FT. (E) Callus composition on day 21 as assessed by histomorphometry. (F) Percentage of mice with bony bridging of the fracture gap. (G–I) Representative histological sections of fractured femurs 21 days after osteotomy stained using Giemsa. (G) non-vibrated OVX mouse, (H) non-vibrated OVX mouse with E2 supplementation, (I) vibrated OVX mouse with E2 supplementation. Scale bars: 500 μm. *P<0.05. *n*=8.

With respect to fracture healing, oestrogen supplementation significantly improved callus properties compared with oestrogen-untreated OVX mice (Fig. 7A–H). Flexural rigidity (+1439%) and BV/TV (+254%) were increased in the oestrogen group (Fig. 7A,C). Consistent with these findings, 50% of the supplemented mice displayed complete cortical bridging, whereas most of the non-supplemented OVX mice were ‘not healed’ (Fig. 7F). Thereby, supplemented mice almost reached the bridging scores of the non-OVX mice (Fig 2F; Fig. 7F), suggesting that oestrogen treatment improved OVX-induced impaired fracture healing.

LMHFV of oestrogen-supplemented OVX mice did not provoke a positive effect on fracture healing (Fig. 7A–I). Neither flexural rigidity nor callus composition was significantly altered compared with non-vibrated oestrogen-supplemented OVX mice (Fig. 7). Notably, LMHFV considerably reduced cortical bridging compared with non-vibrated oestrogen-supplemented OVX mice (Fig. 7F). In conclusion, vibration did not improve fracture healing in oestrogen-supplemented OVX mice, but rather counteracted the positive oestrogen effect.

## DISCUSSION

Because of the proposed osteoanabolic effects of whole-body vibration, we investigated the effect of LMHFV on the OVX-induced impairment of fracture healing. LMHFV significantly improved the compromised fracture healing in OVX mice, and this effect was abolished by external oestrogen application. In OVX mice fracture callus, vibration significantly upregulated ERα, whereas ERβ was unaffected, indicating that ERα might mediate the osteoanabolic response in OVX mice. In contrast, LMHFV considerably impaired non-OVX mice fracture healing. In the fracture callus of these mice, vibration increased ERβ and *Sost* expression, whereas β-catenin levels were decreased, suggesting that ERβ might mediate the negative effects by inhibiting osteoanabolic Wnt/β-catenin signalling.

To characterise our mouse model, we first analysed the effect of OVX on intact bone and fracture healing. As shown previously (Bouxsein et al., 2005; Wright et al., 2008), OVX induced an osteoporotic phenotype with a significant decline in trabecular and cortical bone properties. This effect was more pronounced in the femur compared with the lumbar spine. Such site-specific effects are known and can be explained by local differences in bone metabolic activity (Bouxsein et al., 2005; Liu et al., 2014). In agreement with our own previous data (Beil et al., 2010) and other studies (Namkung-Matthai et al., 2001; Oliver et al., 2013), fracture healing was considerably disturbed after OVX through impaired intramembranous and endochondral bone formation. On the molecular level, we, like others (He et al., 2011), found upregulated callus ERβ mRNA and protein levels, whereas ERα expression was unaffected by OVX. Chondroblasts in particular stained intensively for ERβ, indicating that ERβ signalling might play a role in the prolonged persistence of cartilage residuals. This is supported by the accelerated endochondral ossification and improved bone healing in ERβ-null mice (He et al., 2012).

The ER and Wnt signalling pathways interact at various levels in regulating bone homeostasis (Baron and Kneissel, 2013). ERs regulate *Sost* expression (Bhukhai et al., 2012; Galea et al., 2013), the gene encoding sclerostin, a negative regulator of osteoblast activity produced by osteocytes. Sclerostin antagonises Wnt binding to its low-density lipoprotein receptor-related protein-5 and -6 co-receptors, thereby inhibiting Wnt/β-catenin signalling and osteoblast activity (Semenov and He, 2006). Notably, we observed upregulated *Sost* expression and more sclerostin-stained osteocytes in the fracture callus, indicating Wnt signalling inhibition, which was further corroborated by the reduced β-catenin immunostaining in chondrocytes and osteoblasts. Because Wnt/β-catenin signalling is crucial for bone formation and fracture healing (Baron and Kneissel, 2013), its inhibition might play an important role in the pathomechanisms of impaired healing following OVX. In agreement with these results, we observed increased *Tnfsf11* expression, encoding for RANKL, a stimulator of osteoclast activity, but a downregulation of *Tnfrsf11b*, a target gene of canonical Wnt signalling encoding the RANKL antagonist OPG (Glass et al., 2005). The altered OPG:RANKL ratio in the fracture callus of OVX mice accounts for increased osteoclast activity, which has been described by others in OVX-associated impaired fracture healing (Chung et al., 2012). The expression of other well-established osteogenic (*Bglap* and *Spp1*) and chondrogenic (*Col2a1* and *Sox9*) markers were unaffected. Nevertheless, our data indicate that an altered ERα:ERβ expression ratio might contribute to the bone healing impairment after OVX through inhibition of Wnt/β-catenin signalling.

We have previously demonstrated that LMHFV moderately reduces bone formation in the fracture callus of 12-week-old non-OVX mice (Wehrle et al., 2014) when using a vibration protocol that was osteoanabolic in the intact mouse skeleton (Xie et al., 2006). Here, we demonstrated that vibration was even more harmful in aged non-OVX mice. Vibration strongly inhibited bone formation and impaired the mechanical competence of the callus, while the fracture gap was not bridged with bone. Interestingly, on the molecular level, the vibration-induced effects were similar to those induced by OVX. Vibration led to upregulated ERβ gene expression in the callus of the non-OVX mice, confirmed using immunostaining, whereas ERα expression was unaffected. The expression of ERs is regulated by mechanical stimuli (Lau et al., 2006; Zaman et al., 2010), and ERs regulate load-induced bone adaptation in a complex manner involving both ligand-dependent and -independent mechanisms (Galea et al., 2013). Evidence suggests that ERα and ERβ might have opposing effects in mechanotransduction. Because mechanically induced periosteal bone formation is more effective in female ERβ-null mice, it has been suggested that ERβ might inhibit bone formation in response to mechanical loading (Saxon et al., 2007; Saxon and Turner, 2006). Moreover, low-dose oestrogen treatment suppresses periosteal bone formation in response to mechanical loading (Saxon and Turner, 2006). In contrast, signalling through ERα mediates anabolic effects in mechanotransduction (Jessop et al., 2001; Lee et al., 2003; Saxon and Turner, 2005). Therefore, we suggest that the vibration-induced ERβ increase might account for the inhibited bone formation in non-OVX mice fracture calluses. Furthermore, ER and Wnt signalling have been shown to interact during mechanotransduction (Galea et al., 2013; Liedert et al., 2010). This was confirmed in our model by the increase in ERβ, which was associated with a significant upregulation of *Sost* and a decrease in β-catenin, indicating inhibition of osteoanabolic Wnt/β-catenin signalling, similar to that in OVX-induced impaired fracture healing.

Notably, vibration had contrasting effects in aged-matched OVX mice, whereby LMHFV significantly improved fracture healing compared with non-vibrated OVX mice. Our results confirm, at least in part, those in a previous report showing that LMHFV did not have any effect in older non-OVX rats but improved bone healing in aged-matched OVX rats (Shi et al., 2010). Those authors speculated that, in bone healing, low oestrogen levels might be associated with high mechanosensitivity and that an altered expression of ERs might mediate the LMHFV effects. Our molecular data support this assumption. ERβ expression in the fracture callus, which was enhanced both by OVX and vibration compared with non-OVX mice, was not further increased in vibrated OVX animals. In contrast, ERα expression was significantly upregulated in the callus homogenates, as visualised by immunostaining. Taken together, in the absence of oestrogen, vibration induced a shift in the ERα:ERβ ratio, which could account for the improved bone healing, due to the above-mentioned proposed opposing effects of both receptors in mechanotransduction (Jessop et al., 2001; Saxon et al., 2007; Saxon and Turner, 2005; Saxon and Turner, 2006). We also observed significant upregulation of osteocalcin mRNA and protein, indicating enhanced osteoblast differentiation.

To confirm the crucial role of oestrogen in the distinct vibration-induced effects in non-OVX and OVX mice, we supplemented oestrogen using subcutaneous 17β oestrogen pellets. We hypothesised that, if oestrogen was a key player in the mechanostimulation of fracture healing, it would abolish or at least diminish the positive LMHFV effects. Although oestrogen treatment returned uterus weight and oestrogen serum levels to physiological values, the OVX-induced bone loss was overcompensated, at least in the trabecular bone of the distal femur. Trabecular number and thickness were significantly increased, exceeding that of aged non-OVX and young mice (unpublished data). It has also been demonstrated by others that external oestrogen application does not completely reverse the physiological bone status in mice (Lane et al., 1999; Modder et al., 2004; Otto et al., 2012), which might be a limitation of this model. As expected from the literature (Kolios et al., 2010; Stuermer et al., 2010b), oestrogen treatment improved the OVX-induced compromised fracture healing. Bending stiffness, callus volume, bone formation and bony bridging of the fracture gap significantly increased, and the cartilage fraction decreased, although healing did not reach the level of age-matched non-OVX mice. As hypothesised, LMHFV did not further improve fracture healing in the oestrogen-supplemented OVX mice. Healing parameters remained below non-OVX controls, whereas in OVX mice without oestrogen supplement, vibration fully compensated for compromised fracture healing. Taken together, these results confirm the key role of oestrogen in the vibration-induced effects on fracture healing.

Taken together, LMHFV disturbed fracture healing in aged mice but improved it in age-matched OVX mice, indicating that the oestrogen status plays a major role in the mechanostimulation of fracture healing. Our molecular results imply that the LMHFV-induced osteoanabolic effects in OVX mice might be mediated by ERα-regulated gene transcription, whereas ERβ signalling might be responsible for inhibited bone formation in vibrated non-OVX mice. Transferring the present results to a clinical setting, LMHFV would be an attractive therapy to stimulate bone healing in osteoporotic patients. However, the distinct oestrogen-dependent effects imply that LMHFV might only be safe and effective in elderly postmenopausal osteoporotic women. Further pre-clinical studies beyond rodent models are mandatory, and caution must be paid in clinical trials to restrict application of the technique to suitable patients.

## MATERIALS AND METHODS

### Animals

All animal experiments were performed in compliance with the guide for the care and use of laboratory animals (National Research Council, 2011) and were approved by the local ethical committee (No. 1026 and 1113, Regierungspräsidium Tübingen, Germany). Female C57BL/6NCrl mice (*n*=96) were purchased from Charles River (Sulzfeld, Germany). The mice received a standard mouse feed (ssniff® R/M-H, V1535-300, Ssniff, Soest, Germany). At 2 weeks prior to OVX, the diet of the respective animals was changed to a phytooestrogen-reduced chow (ssniff^®^ R/M-H Phytooestrogenarm, Ssniff).

### Study design

The mice were randomly assigned to six groups (Table 1). At age 41 weeks the mice were either bilaterally ovariectomised (OVX, groups 3–6) or sham-operated (non-OVX, groups 1 and 2). Immediately after OVX, subcutaneous 17β oestrogen pellets (0.025 mg per pellet, 90 day-release; Innovative Research of America, Sarasota, FL) were implanted in a subset of 16 mice (groups 5 and 6). When aged 49 weeks, all mice received a femur osteotomy and were randomly assigned to non-vibrated control (non-LMHFV) or treatment groups (LMHFV) (Table 1). The treatment groups received LMHFV as described below. The mice were killed at 10 (*n*=5–7 in groups 1–4) or 21 days (*n*=8 in groups 1–6) after surgery. Intact bones (contra-lateral femurs, lumbar spine) and the osteotomised femurs were evaluated using biomechanical testing, μCT and histomorphometry. On day 10, callus homogenates of groups 1–4 (*n*=6) were used to assess gene expression using a quantitative polymerase chain reaction (qPCR).

### Femur osteotomy

Surgery was performed as described previously (Wehrle et al., 2014). Briefly, a 0.4-mm osteotomy gap was created at the mid-shaft of the right femur and stabilised using an external fixator (axial stiffness 3 N/mm, RISystem, Davos, Switzerland). Preoperatively, all mice received a single dose of antibiotic (clindamycin-2-dihydrogenphosphate, 45 mg/kg of body weight, Clindamycin, Ratiopharm, Ulm, Germany). Analgesia (25 mg/l, Tramal^®^, Gruenenthal GmbH, Aachen, Germany) was provided through the drinking water 2 days before and 3 days after surgery.

**Table 1.**
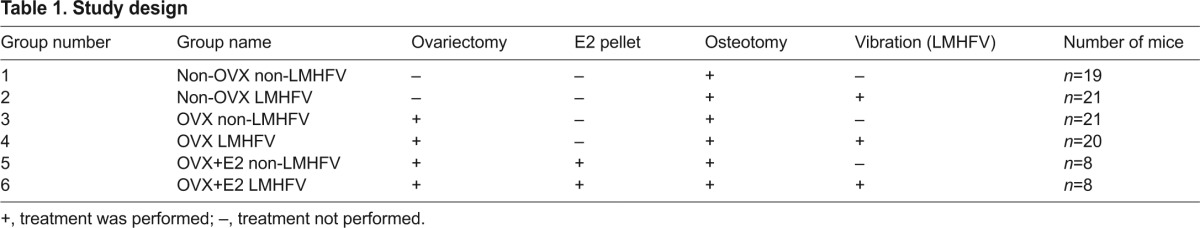
Study design

### LMHFV treatment

LMHFV was applied using custom-made vibration platforms as described previously (Wehrle et al., 2014). Starting on the third postoperative day, the mice were placed on the platforms for 20 minutes per day for 5 days per week and received vertical vibration with 0.3 ***g*** peak-to-peak acceleration (*a*_peak-to-peak_) and a frequency (*f*) of 45 Hz. Controls were sham vibrated.

### Biomechanical testing

The mechanical properties of intact and osteotomised femurs explanted on day 21 were investigated using a non-destructive three-point bending test and flexural rigidity was calculated as described previously (Wehrle et al., 2014).

### μCT

On day 21, femurs and lumbar vertebrae were scanned in a μCT device (Skyscan 1172, Kontich, Belgium) at 8 μm resolution using a voltage of 50 kV and 200 μA. Calibration and global thresholding (641.9 mg hydroxyapatite/cm^3^ for callus and cortical bone; 394.8 mg hydroxyapatite/cm^3^ for trabecular bone) was performed as described previously (Wehrle et al., 2014). Non-fractured femurs [trabecular volume of interest (VOI): 90 slices of 8-μm thickness in distal femur; cortical VOI: 250 slices of 8 μm], lumbar spine (trabecular bone: spherical VOI, d=0.8 mm) and the osteotomy gap were evaluated. Common ASBMR standard parameters were determined (Dempster et al., 2013). According to the standard clinical evaluation of X-rays, the number of bridged cortices per callus was evaluated in two perpendicular planes (Data viewer, Skyscan, Kontich). A ‘healed fracture’ was considered to have ≥3 bridged cortices per callus.

### Histomorphometry

The bone specimens were embedded in paraffin (day 10) or methyl methacrylate (day 21). Safranin O (day 10; *n*=5–7 per group) or Giemsa (day 21; *n*=8 per group) staining was performed on longitudinal sections of the fracture calli. In the region of interest (ROI), the relative amounts of newly formed bone (TOT), cartilage (Cg) and fibrous tissue (FT) were determined using image analysis (Leica DMI6000 B; Software MMAF Version 1.4.0 MetaMorph^®^; Leica, Heerbrugg, Switzerland). On day 10, the periosteal callus between the inner pins of the fixator and on day 21 the osteotomy gap was defined as the ROI (Wehrle et al., 2014).

### Immunohistochemistry

Immunohistochemical staining for ERs (ERα: sc-542; ERβ: sc-8974, Santa Cruz Biotechnology, Dallas, TX), sclerostin (AF1589, R&D Systems, Minneapolis, MN), osteocalcin (orb 77248, Biorbyt Ltd, Cambridge, UK) and β-catenin (06-734, Merck Millipore, Billerica, MA) was performed on paraffin-embedded sections after deparaffinisation using xylene and rehydration using methanol. After blocking nonspecific sites [ERα and ERβ: phosphate-buffered saline (PBS) + 10% goat serum; sclerostin: PBS + 10% horse serum; osteocalcin: tris-buffered saline (TBS)-Triton X-100 + 5% goat serum; β-catenin: 4% BSA + 0.1% Triton X-100] for 60 minutes at room temperature, the sections were incubated with the primary antibody against ERα (1:75 in PBS + 2% goat serum), ERβ (1:40 in PBS + 1% goat serum), sclerostin (1:200 in PBS + 1% horse serum), osteocalcin (1:200 in TBS-Tween 20 + 1% goat serum) or β-catenin (1:150 in PBS + 1% BSA + 0.1% Triton-X) overnight at 4°C. To detect the primary antibody, either a secondary biotinylated goat anti-rabbit-IgG antibody (Invitrogen, Life Technologies Corporation, Darmstadt, Germany; for ERα, ERβ, osteocalcin and β-catenin) or a donkey anti-goat-IgG antibody (Invitrogen, Life Technologies Corporation; for sclerostin) was added for 30 minutes at room temperature. For signal amplification, the slides were incubated either with streptavidin-conjugated horseradish peroxidase (Zytomed Systems, Berlin, Germany) for 15 minutes (ERα, sclerostin and β-catenin) or avidin-biotin complex (Vector, Burlingame, CA) for 30 minutes (ERβ and osteocalcin). Chromogen AEC Single Solution (Zytomed) or NovaRed (Vector) was used as the detection substrate. Counterstaining was performed using hematoxylin (Waldeck, Münster, Germany). Species-specific IgG was used as isotype control in an additional section of each sample.

### Gene-expression analysis in the fracture callus

On day 10, fractured femurs (*n*=6 per group) were snap-frozen in liquid nitrogen and stored at −80°C. The callus between the inner pins, including marginal cortical bone, was excised and pulverised in a vibration mill at 30 Hz for 2.5 minutes. After adding 1 ml Trizol, the samples were centrifuged at 5000 ***g*** for 30 seconds and callus RNA was isolated using the RNeasy-Mini-Kit (Qiagen, Hilden, Germany) according to the manufacturer’s instructions, including a DNase digestion step. The RNA was eluted in 30 μl RNase-free water and stored at −80°C. Extracted RNA concentration and integrity were verified using 260 nm spectrophotometry with the reference wavelengths 280 and 230 nm and agarose gel electrophoresis. Reverse transcription of 1 μg of RNA was performed using the Omniscript RT kit (Qiagen). The PCR reaction mixture (12.5 μl SYBR Green MasterMix, 0.25 μl ROX, 0.1 μM forward primer and 0.1 μM reverse primer, 8.25 μl RNase-free water and 2 μl cDNA) was prepared and each sample was tested in duplicates using an Abi StepOne Plus Cycler (Applied Biosystems, Darmstadt, Germany) using the following cycling conditions: 50°C for 2 minutes, 95°C for 2 minutes, 40 cycles each consisting of 95°C for 15 seconds and 60°C for 1 minute (see supplementary material Table S1 for primer sequences). Then, melting curve acquisition was performed (95°C for 15 seconds, 60°C for 1 minute, 95°C for 15 seconds). Data were analysed using the ΔΔC_t_ method. C_t_ values obtained for each sample were normalised to those for the house-keeping gene *GAPDH*; ΔΔC_t_=ΔC_t_(treatment group) – ΔC_t_(control group) was calculated and a fold-change in gene expression of the treatment group versus the control group was expressed as 2^–ΔΔCt^.

### Oestrogen serum levels

Oestrogen serum levels were determined (*n*=6 per group and time point) using a competitive ELISA kit (Oestradiol ELISA, Calbiotech, Spring Valley, CA) with a detection limit of 3.94 pg oestrogen/ml serum.

### Statistical analysis

Sample size was calculated based on previous studies (Wehrle et al., 2014) for two main outcome parameters: flexural rigidity in fractured femurs and trabecular BV/TV in non-fractured femora (power, 80%; alpha, 0.05). Results are presented as the mean ± s.d. Data were tested for normal distribution (Shapiro-Wilk normality test) and homogeneity of variance (Levene test). Dependent on the test outcome, a comparison of either non-OVX versus OVX, OVX versus OVX+E2 or non-LMHFV versus LMHFV groups was made using a Student’s *t*-test (normally distributed data with homogenous variance) or the Mann–Whitney U-test (data either not normally distributed and/or not showing homogenous variance; IBM SPSS Statistics Version 19). The level of significance was set at *P*≤0.05.

## Supplementary Material

Supplementary Material
