# Supplementary material for: The impact of low-magnitude high-frequency vibration on fracture healing is profoundly influenced by the oestrogen status in mice
Source: Dis Model Mech. 2014 Nov 7;8(1):93–104. doi: 10.1242/dmm.018622 (PMC4283653; doi:10.1242/dmm.018622)
Supplement: Supplementary Material [file supp_8_1_93__index.html]

The impact of low-magnitude high-frequency vibration on fracture healing is profoundly influenced by the oestrogen status in mice — Supplementary Material 

# The impact of low-magnitude high-frequency vibration on fracture healing is profoundly influenced by the oestrogen status in mice

## DMM018622 Supplementary Material

**Files in this Data Supplement:**

- **Supplementary Material**
