# Supplementary material for: The impact of low-magnitude high-frequency vibration on fracture healing is profoundly influenced by the oestrogen status in mice
Source: Dis Model Mech. 2014 Nov 7;8(1):93–104. doi: 10.1242/dmm.018622 (PMC4283653; doi:10.1242/dmm.018622)
Supplement: Supplementary Material [file supp_8.1.93_DMM018622.pdf]

Table S1. Primer sequences

Target gene	Forward primer (5′-3′)	Reverse primer (5′-3′)	NCBI Acc. No.	Product size (bp)
Bglap	gcg ctc tgt ctc tct gac ct	acc tta ttg ccc tcc tgc tt	NM_007541	89
cmyc	ccc caa ggt agt gat cct ca	tcg tct gct tga atg gac ag	NM_010849	60
Col2a1	cct gtc tgc ttc ttg taa aac	tgg gta tca tca ggt cag gt	NM_031163	134
Esr1	tcc ggc aca tga gta aca aa	cca gga gca ggt cat aga gg	NM_007956	84
Esr2	gag tag ccg gaa gct gac ac	tct tca aaa tca ccc aga cc	NM_207707	68
Gapdh	aac ttt ggc att gtg gaa gg	cac att ggg ggt agg aac ac	BC083149	81
Sost	atg acg cca aag atg tgt cc	gtc agg aag cgg gtg tag tg	NM_024449	61
Sox9	ctc tgg gca atc tca ggg tcc	aac ttt gcc agc ttg cac gt	NM_011448	70
Spp1	cga tga tga tga cga tgg ag	cag att cat ccg agt cca ca	AF515708	62
Tnfrsf11b	ctg cct ggg aag aag atc ag	gct cga ttt gca ggt ctt tc	MMU94331	63
Tnfsf11	atc atg aaa cat cgg gaa gc	ctt ggg att ttg atg ctg gt	AF019048	82
